# Westminster Hospital

**Published:** 1831-02

**Authors:** 


### HOSPITAL REPORTS.

### WESTMINSTER HOSPITAL.

Two cases of amputation have recently occurred at this hospital.

Case I. James Lansdown, aged seven, admitted for a scrofulous disease of the ankle-joint. Amputation was performed by Sir Anthony Carlise, by the flap operation, on the 6th November. Secondary hemorrhage took place in the same evening, and about fourteen ounces of blood were lost before the artery was secured. Nothing untoward occurred after this, and the child went on well to his recovery.

Case II. John Bellis, aged twenty, admitted August 11th, 1830, with scrofulous disease of the right ankle-joint, excited by a blow inflicted upon it four months back. The part has always been under treatment since the injury: constant rest, with the different methods employed in similar cases, have been tried without any benefit. He is now quite unable to move the joint, and passive motion is exceedingly limited and painful. The surrounding parts are very much swollen and thickened, more than twice their natural size, and there are several ulcerations with callous edges, the openings of sinuses leading into the joint. His health is also a good deal impaired by the constant irritation kept up by the diseased limb, and the want of proper exercise. It was agreed in consultation that amputation was necessary, and the operation was performed the 6th of November, 1830. Mr. Guthrie amputated by a circular incision, about seven inches below the knee. The muscles of the limb were very much wasted, but plenty of integument was saved to form a good stump. The sharp projection formed by the extremity of the anterior spine of the tibia was sawed off. Three arteries only required ligatures, and the stump was then dressed in the usual manner. Very little blood was lost, and the man was not at all faint or nervous during the operation.

Nine P.M. He appears quite comfortable: complains only of some pain in the stump, resembling that lately caused by the diseased joint. He is rather thirsty, but not feverish; skin moist, and not unnaturally warm; pulse 116, and tolerably firm.

Nov. 7th. Mr. Guthrie saw him at two o'clock, and remarked that there was too much pulse and general increased action. As the bowels had not been acted upon since the operation, he ordered Extr. Colocyn. comp. gr. ij., Hydr. Submur. gr. iv., to be taken immediately, and the Infus. Sennæ cum Magnes. Sulph. two hours after; and that if, after the operation of the medicines, the increased action continued unabated, blood was to be taken from the arm.

Ten P.M.— His bowels have not yet been freely opened by the medicines. The general feverishness is much increased; pulse full, 120; there is constant heat of skin, with occasional perspiration. He complains of pains in his chest and abdomen, rather increased on pressure, and on taking a full inspiration. Complains also of slight pain and uneasiness of the stump. Fourteen ounces of blood were taken from the arm, and the bandages were all removed from the stump, and the purgatives were repeated.

8th. The blood drawn yesterday is very much buffed and cupped. He has passed a restless night, and was sick early this morning. He is now, however, better; pulse 130. His bowels were acted upon twice during the night, and once this morning. The pains in his chest and abdomen are less, and the stump is tolerably easy. Mr. Guthrie desired that all the dressings should be removed, and a poultice applied to the stump, the wound in which has nearly united by the first intention in its whole extent, but the surrounding parts are swollen. He was again bled to fourteen ounces, and ordered the following draught every six hours: ℞. Tinct. Digitalis, Tinct. Opii, aa ♏vi.; Mist. Salin. ℥iss. M. fiat haustus.

9th. Slept very little last night. The pains in his chest and abdomen are now very trifling, but he complains of the stump being hot and uneasy. The blood drawn yesterday is a good deal buffed, but not cupped. His tongue is thickly coated; skin hot; pulse sharp and 150; bowels have not been opened since yesterday morning. On removing the poultice, the stump appears rather more inflamed and swollen, and pressure is painful as high as the knee. Fomentations to be constantly applied, and to continue the draughts every four hours. Infus. Sennæ cum Magn. Sulph.

10th. Has passed a bad night, the whole limb having been very uneasy. He complains of constant nausea, and has been sick several times. The digitalis to be discontinued, and the effervescing draught, with opium, to be taken every four hours. There is a small quantity of matter in the poultice this morning. The swelling has extended considerably above the knee, and pressure on any part is painful. The poultice and fomentations to be continued. Bowels open.

11th. Did not sleep last night, but feels better this morning, the sickness having gone off. He is, however, looking very unwell: slight hectic appearance of the countenance, tongue furred, skin hot, pulse continues at 150, and is now rather feeble; bowels open. The wound is suppurating freely. The swelling and inflammation have now reached as high as the trochanter, and the outer and under part of the thigh looking very red. Pressure in the course of the saphena vein is very painful, but the skin is not there looking red. Poultices applied to all these parts. The effervescing draughts, with opium, to be continued.

13th. Passed a tolerably good night, and he is better. Pulse 135, and stronger; tongue rather white, but not furred; bowels open. The limb is less swollen and inflamed, and he can bear pressure better. The face of the stump is suppurating freely.

15th. Is going on well: the swelling and inflammation have nearly disappeared from the thigh, and it is considerably diminished also at the stump; there is no pain on pressure in the course of the vein; his pulse is 100, and about its natural strength; tongue clean; bowels open. Two ligatures came away in the poultice yesterday morning, being the eighth after the operation. The poultices to be continued.

17th. The third ligature has come away this morning: a piece of simple dressing is now to be applied to the wound, which is discharging a good deal of matter. The inflammation has returned in one spot, about the middle of the outer part of the thigh; says that he feels better; pulse continues small and quick, about 100; tongue clean; no appetite. To take the infusion of roses, with acid, every six hours.

19th. The inflammation on the outer part of the thigh is increased, and there is rather a diffused swelling, having a soft fluctuating feel. Mr. Guthrie made a deep incision through the fascia at this spot, and allowed a quantity of matter to escape. A poultice to be applied. ℞. Quin. Sulph. gr. vi., Acid. S. D. ♏xx., Infus. Rosæ ℥vi. sumat cochl. iij. ter die.

20th. A quantity of matter has been discharged from the wound made yesterday, and pressure upwards and downwards causes more to flow from it. This abscess has no communication with the stump. Some matter had also collected on the inner side of the stump: it was pressed out, and a roller applied. He is much better: slept well last night; tongue clean; appetite improved. To have mutton chops for his dinner. Cont. mist.

December 1st. The thigh is now quite free from inflammation, and the wound in the outer part has healed; the wound is rather tender to the touch on the inner side, but the face of it is granulating healthily. Has continued regularly the quinine mixture, and his mutton chops and beef-tea. He is now looking much better; tongue clean, appetite improved, and he sleeps well at night; pulse has always been, since the last report, about ninety, and soft.

10th. He has been out of bed every day during the last week. For the first four days he had rheumatic pains in his back and limbs, so that he was unable to move without being supported: he can now, however, walk easily with his crutches. Complains of general weakness; otherwise, his health is much better than it was before the operation: his pulse is at ninety, and is soft; tongue clean; appetite very good; bowels open. The stump is healing up rapidly, but is rather tender to the touch at the inner part, where a puncture has been made. Yesterday there was a slight return of the redness and tension of the thigh, but it is now quite gone. To continue the mixture, and the mutton chops and porter.

16th. The stump is all but well, and his health is good.

